# Differential Expression of microRNAs in the Non-Permissive Schistosome Host *Microtus fortis* under Schistosome Infection

**DOI:** 10.1371/journal.pone.0085080

**Published:** 2013-12-31

**Authors:** Hongxiao Han, Jinbiao Peng, Yanhui Han, Min Zhang, Yang Hong, Zhiqiang Fu, Jianmei Yang, Jianping Tao, Jiaojiao Lin

**Affiliations:** 1 Shanghai Veterinary Research Institute, Chinese Academy of Agricultural Sciences, Key Laboratory of Animal Parasitology, Ministry of Agriculture, Minhang, Shanghai, China; 2 Shanghai Public Health Clinical Center, Fudan University, Shanghai, China; 3 College of Veterinary Medicine, Yangzhou University, Yangzhou, Jiangsu, China; 4 Jiangsu Co-innovation Center for Prevention and Control of Important Animal Infectious Diseases and Zoonoses, Yangzhou, China; Uppsala University, Sweden

## Abstract

The reed vole *Microtus fortis* is the only mammal known in China in which the growth, development and maturation of schistosomes (*Schistosoma japonicum*) is prevented. It might be that the anti-schistosomiasis mechanisms of *M. fortis* associate with microRNA-mediated gene expression, given that the latter has been found to be involved in gene regulation in eukaryotes. In the present study, the difference between pathological changes in tissues of *M. fortis* and of mice (*Mus musculus*) post-schistosome infection were observed by using hematoxylin-eosin staining. In addition, microarray technique was applied to identify differentially expressed miRNAs in the same tissues before and post-infection to analyze the potential roles of miRNAs in schistosome infection in these two different types of host. Histological analyses showed that *S. japonicum* infection in *M. fortis* resulted in a more intensive inflammatory response and pathological change than in mice. The microarray analysis revealed that 162 miRNAs were expressed in both species, with 12 in liver, 32 in spleen and 34 in lung being differentially expressed in *M. fortis*. The functions of the differentially expressed miRNAs were mainly revolved in nutrient metabolism, immune regulation, etc. Further analysis revealed that important signaling pathways were triggered after infection by *S. japonicum* in *M. fortis* but not in the mice. These results provide new insights into the general mechanisms of regulation in the non-permissive schistosome host *M. fortis* that exploits potential miRNA regulatory networks. Such information will help improve current understanding of schistosome development and host–parasite interactions.

## Introduction

Schistosomiasis is one of the most serious zoonotic diseases, afflicting more than 200 million individuals in tropical and sub-tropical regions [1]. It is caused by trematodes of the genus *Schistosoma*, the most prevalent species being *Schistosoma mansoni*, *Schistosoma japonicum* and *Schistosoma haematobium* [2]. It is reported that more than 46 species of mammals are naturally infected by *S. japonicum* in China. The reed vole, *Microtus fortis* is the only mammal found in endemic areas of China in which the parasite is naturally prevented from maturing and completing its life cycle [3,4,5]. Most of the worms are consumed in the lung and those that remain migrate to the liver, where they are then eliminated; thus, no adult worms or eggs have been found in any schistosome-infected *M. fortis* [6]. By contrast, BALB/c mice are a susceptible host for *S. japonicum* and approximately 70% of worms are able to complete their life cycle in this animal. Previous reports indicate that humoral and/or cellular immunity might have an important role in the restricted development of *S. japonicum* in *M. fortis*. Some studies have been carried out to investigate the mechanisms and immunopathogenesis behind the host response to schistosome infection in liver, lung and spleen [7,8,9]. Jiang et al. reported that some immune-related and apoptosis-inducing genes were up-regulated, whereas some development-associated genes were down-regulated in *M. fortis* 10 days post-infection compared with uninfected animals. These results suggest that different hosts have different response mechanisms to schistosome infection [10].

MicroRNAs (miRNAs) are a class of endogenous, small noncoding RNAs that modulate gene expression at the post-transcriptional level. miRNAs by binding to their target mRNAs, causing a block of translation or degradation of mRNA [11]. MiRNAs are sequentially processed from primary transcripts (termed ‘pri-miRNAs’) into approximately 70-nucleotide (nt) stem-loop precursors in the nucleus, and then further cleaved in the cytoplasm by the Dicer enzyme into approximately 23-nt functional sequences [12]. Since Ambros et al. reported the discovery of the first miRNA, lin-4 in *Caenorhabditis elegans* [13], it has been further confirmed that miRNAs are evolutionarily conserved in many species, demonstrating their universal roles in the regulation of gene expression. It has been shown that miRNAs have fundamental roles in diverse biological and pathological processes, including development, apoptosis, proliferation, differentiation, organ development, carcinogenesis, energy metabolism, and the immune response [14,15,16]. In recent years, studies have shown that miRNAs are deregulated under different pathological conditions, such as cancer and liver injury. MiRNA expression profiles have also been reported for distinguishing cancerous from non-cancerous tissue for finding biomarkers or therapeutic targets [17,18]. MiRNA might also be an important factor in the complex interaction between parasites and their hosts, as well as in parasite drug resistance [19,20,21]. A class of miRNAs was found to regulate promoter binding of the nuclear factor (NF)-kB p65 subunit in human cholangiocytes in response to *Cryptosporidium parvum* infection, which might be relevant to the regulation of epithelial antimicrobial defense [21]. However, only a few studies have investigated the differences in miRNA expression and its specific biological function(s) in hosts infected by parasites [22,23]. 

In the current study, the inflammatory response and pathological changes in different tissues from *M. fortis* were observed using hematoxylin-eosin staining. In addition, specific differential expression of miRNAs in *M. fortis* was identified by using miRNA microarray technique. The biological functions of the differentially expressed miRNAs were investigated by bioinformatics analysis. The results provide useful comparative information to better define the function of miRNAs during the infection of *M. fortis* by *S. japonicum* and could lead to a better understanding of schistosome development and host–parasite interactions.

## Materials and Methods

### Animal challenge and tissue preparation

Six-week-old specific pathogen-free (SPF) male BALB/c mice (each weighing approximately 20 g) and male *Microtus fortis* (each approximately 60 g) were obtained from the Shanghai Laboratory Animal Center, Chinese Academy of Sciences and Shanghai *Xipu*'*er-bikai* Experimental Animal Co., Ltd (Shanghai), respectively. All animal care and experimental procedures were conducted according to the guidelines for animal use in toxicology (Society of Toxicology USP, 1989). The study protocol was approved by the Animal Care and Use Committee of the Shanghai Veterinary Research Institute, Chinese Academy of Agricultural Sciences. For the experiment, 30 *M. fortis* and 30 BALB/c mice were subdivided into three groups of 10 each. Three additional animals were used as uninfected controls. The infection experiment was repeated in three independent biological replicates. All animals were singly housed for 1 week before infection. Food and water was available ad libitum. *M. fortis* and BALB/c mice were percutaneously infected with 3000 and 200 *S. japonicum* cercariae (Chinese mainland strain, Anhui isolate), respectively. The animals were sacrificed at 10 days post-infection (p.i.) and the lung, liver and spleen were harvested and preserved in RNAlater® (Ambion) at –80°C until RNA extraction. Tissues collected from the same animals were used for histological analyses.

### Histological assessment

The harvested liver, spleen and lung were dissected after the worms were perfused with phosphate buffered saline (PBS). The tissues were then fixed in 10% formalin (Sigma) and embedded in paraffin wax. Sections were processed and stained by hematoxylin and eosin (H&E) to assess tissue structure. The slides (thickness, 5 um) were visualized using fluorescence microscopy (Nikon, Japan). The image acquisition parameters and microscope settings were kept the same throughout the process.

### Total RNA isolation and quality control

Total RNA extraction from tissues was performed with the mirVana isolation kit, according to the manufacturer’s instructions (Ambion, USA). The quality of RNA was measured using a Nanodrop-1000 and the integrity was evaluated by Agilent 2100 Bioanalyzer (Agilent Technologies, USA). Only cases with RNA Integrity Numbers (RIN) ≥7–10 were used for further study. Briefly, the assay began with 5 ug of RNA from each sample, which was size fractionated using an YM-100 Microcon filter (Millipore, Bedford, MA, USA). The small RNAs (<300 nt) extracted were 3′ extended with poly(A) polymerase. 

### Microarray analysis

MiRNA microarrays following the miRbase v17.0 were used to study the expression profiling of 1777 mature miRNAs, which comprised 1096 miRNAs in mouse, 679 in rat, two in Chinese hamster and 55 control miRNA sequences. Briefly, hybridizations were started according to the manual using a μParaflo^@^ microfluidic technology (LC Sciences, USA). The Cy5 dye-labeled small RNAs (<300nt) were dissolved in 100 ul 6xSSPE buffer (0.90 M NaCl, 60 mM Na_2_HPO4, 6 mM EDTA, pH 6.8) containing 25% formamide at 34°C overnight. Hybridization images were scanned using a laser scanner (GenePix 4000B, Molecular Device) and digitize analysis was performed using Array-Pro software (Media Cybernetics, Bethesda, MD). Microarray hybridizations were performed in duplicate for all samples. The data were then normalized using a cyclic LOWESS (Locally-weighted Regression) method for further analysis. Full details of this miRNA microarray are deposited in the Gene Expression Omnibus (GEO; http://www.ncbi.nlm.nih.gov/geo/) public database with an associated platform accession number GPL15710. The raw data are available through GEO with the series accession number GSE38802. The entire microarray data are MIAME compliant.

We defined the differentially expressed miRNA using the ratio of detected signals log_2_-fold changes [log_2_(infected/control)] and the Student’s t-test was used to calculate *P* values. The differentially expressed miRNAs were selected based on a fold change >2 or < -2 and P values <0.05. 

### Predicted gene targets of differentially expressed miRNAs, gene ontology and KEGG pathway analysis

Targets of the miRNAs were predicted by using the online software, TargetScanS (http://genes.mit.edu/tscan/targetscanS2005.html) in conjunction with miRanda (http://www.microrna.org/microrna), PicTar (http://pictar.org/) and RNAhybrid (http://140.109.42.4/cgi-bin/RNAhybrid/RNAhybrid.cgi). The target genes of differentially expressed miRNAs were analyzed in terms of their Gene Ontology (GO) categories and Kyoto Encyclopedia of Genes and Genomes (KEGG) pathways, by using the DAVID (Database for Annotation, Visualization and Integrated Discovery) gene annotation tool ( http://david.abcc.ncifcrf.gov/) [24].

### MiRNA–gene network analysis

The top 20% miRNA target genes were collected, and subjected to miRNA–gene network analysis. The relationship between miRNAs and genes was evaluated by their differential expression values, and a miRNA–gene network was constructed according to the interactions of the miRNA and genes in the Sanger miRNA database [25]. The adjacency matrix of miRNA and genes (A=[ai,j]) was established based on the attribute relationships between the genes and the miRNA, where ai,j shows the weight of the relation of gene I with miRNA j. In the diagram of the miRNA–gene network, the circle represents the gene and the square the miRNA, with the relation between them represented by a line. 

Degrees in the center of the network represent the individual contribution of one miRNA or gene to the genes or miRNAs surrounding them. The key miRNAs and genes in the network usually had the highest degree [26,27]. Based on the miRNA degree, the network that represented the crucial miRNAs and their targets could be established.

### Validation of microarray data with qPCR analysis

Differentially expressed miRNAs were examined using quantitative stem-loop reverse transcription RT-PCR (*qPCR*) with SYBR green. The stem-loop reverse transcription primers were designed following the method described by Chen et al [28]. U6 RNA was selected as a housekeeping miRNA to normalize the miRNA expression[29,30,31].The RNA templates for the qPCR were performed on the same samples used for microarray hybridizations. The primers for the qPCR experiment were optimized by the PCR analysis to evaluate the specificity and sensitivity. Total RNA from tissues were quantified by nanodrop-1000 and reverse-transcribed to cDNA using RT primers and a SuperScript^TM^ Ⅲ Reverse Transcriptase kit (Invitrogen, USA). The 25μL qPCR reaction was as follows: 12.5μL SYBR^@^ Premix Ex Taq^TM^II (TaKaRa, Dalian, China), 1μL of forward and reverse primers mixtures, 1μL cDNA template, 0.5μL Rox Reference Dye II and 10μL Easy Dilution. The cycling protocol was as follows: 95°C for 30 sec, followed by 40 cycles of 95°C for 5 sec and 60°C for 34 sec. The quantification of each miRNA relative to U6 was calculated using the 2^-△△Ct^. method. All assays were performed in triplicate. The primer sequences are shown in Table S1.

### Statistical analysis

Data are expressed as mean ± standard deviation (SD). Differences between groups were determined by Student’s *t*-test. Statistical significance was reached at p<0.01.

## Results

### Histological changes in the liver, spleen and lung of *M. fortis* infected with *S. japonicum*

**Figure 1:**
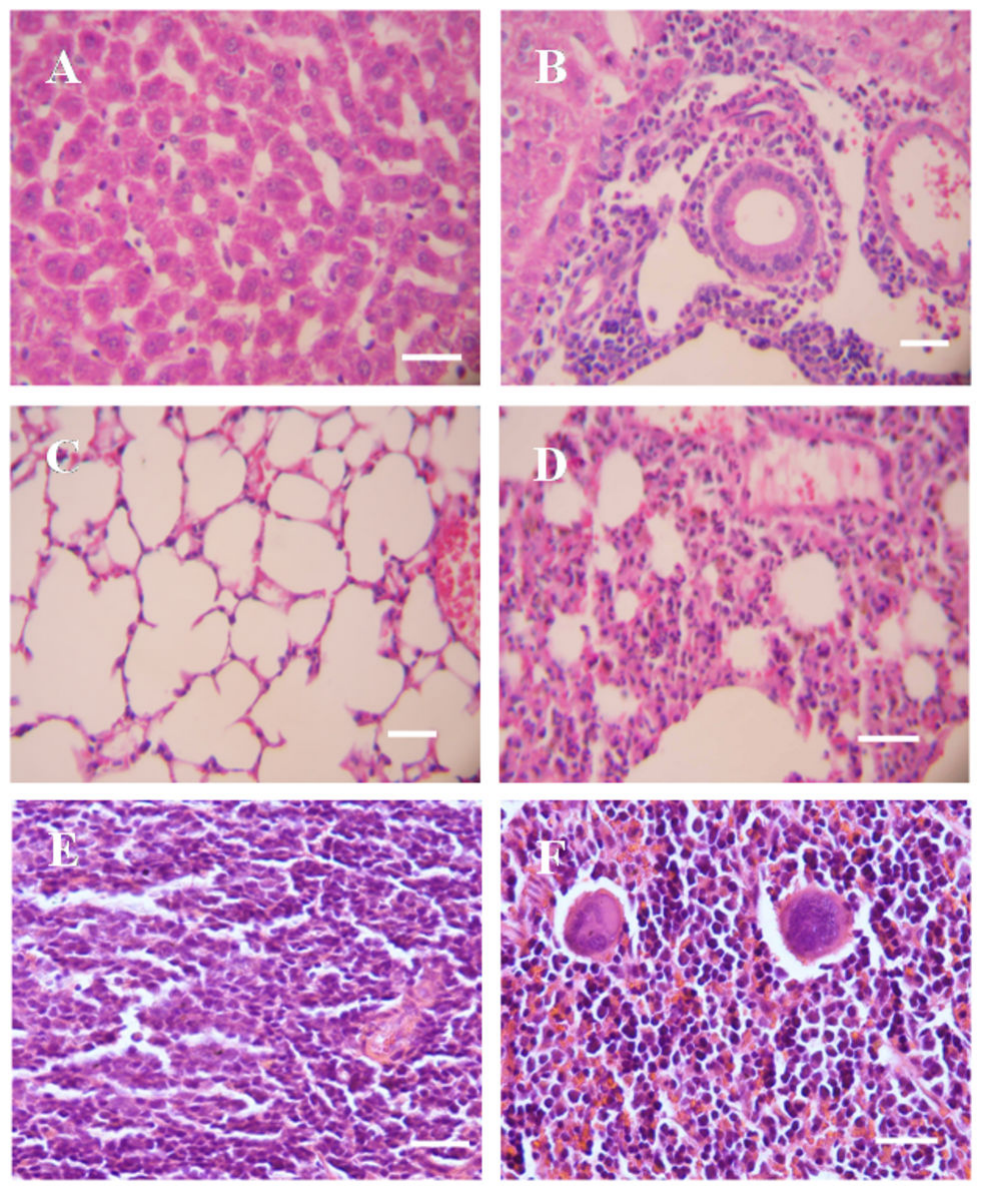
Histopathology of different tissue sections prepared from *M. fortis* by HE staining. (A-F) tissues prepared from *M.fortis* (×400),(A) uninfected liver, (B) infected liver, (C) uninfected lung, (D) infected lung, (E) uninfected spleen, (F) infected spleen, macrophages were observed. Bar=20μm.

Some histological analyses of the liver and lung of *S. japonicum*-infected *M. fortis* and mice have been reported previously [10]. Livers from uninfected control *M. fortis* showed a normal structure (Figure 1A). In the livers of infected *M. fortis*, the edges of the partial hepatic lobule were undefined and some hepatocytes were out of shape and shrinking. A large number of eosinophils and lymphocytes, and a few neutrophils were observed in small inflammatory infiltrates. The bile canaliculi epithelium cells were proliferated and many eosinophils and lymphocytes were present in small inflammatory infiltrates adjacent to the portal area. Some neutrophils were detected on the edges of the hepatic lobule (Figure 1B).In the lungs of infected *M. fortis*, severe pathological damage and many inflammatory cells were observed. The structure of the lungs was destroyed, and the number of eosinophils, macrophages and lymphocytes increased significantly. Some neutrophils were observed in big inflammatory infiltrates compared with uninfected controls, which was concordant with previous reports. Severe hemorrhaging, hemostasis and hemosiderin deposition were observed around the bronchiole (Figure 1C, D).The splenic architecture showed no changes during the early period of *S. japonicum* infection, but more macrophages were observed in infected spleen of *M. fortis* (Figure 1E, F).

### Microarray analysis of differentially expressed miRNAs in tissues in infected *M. fortis* and mice

**Figure 2:**
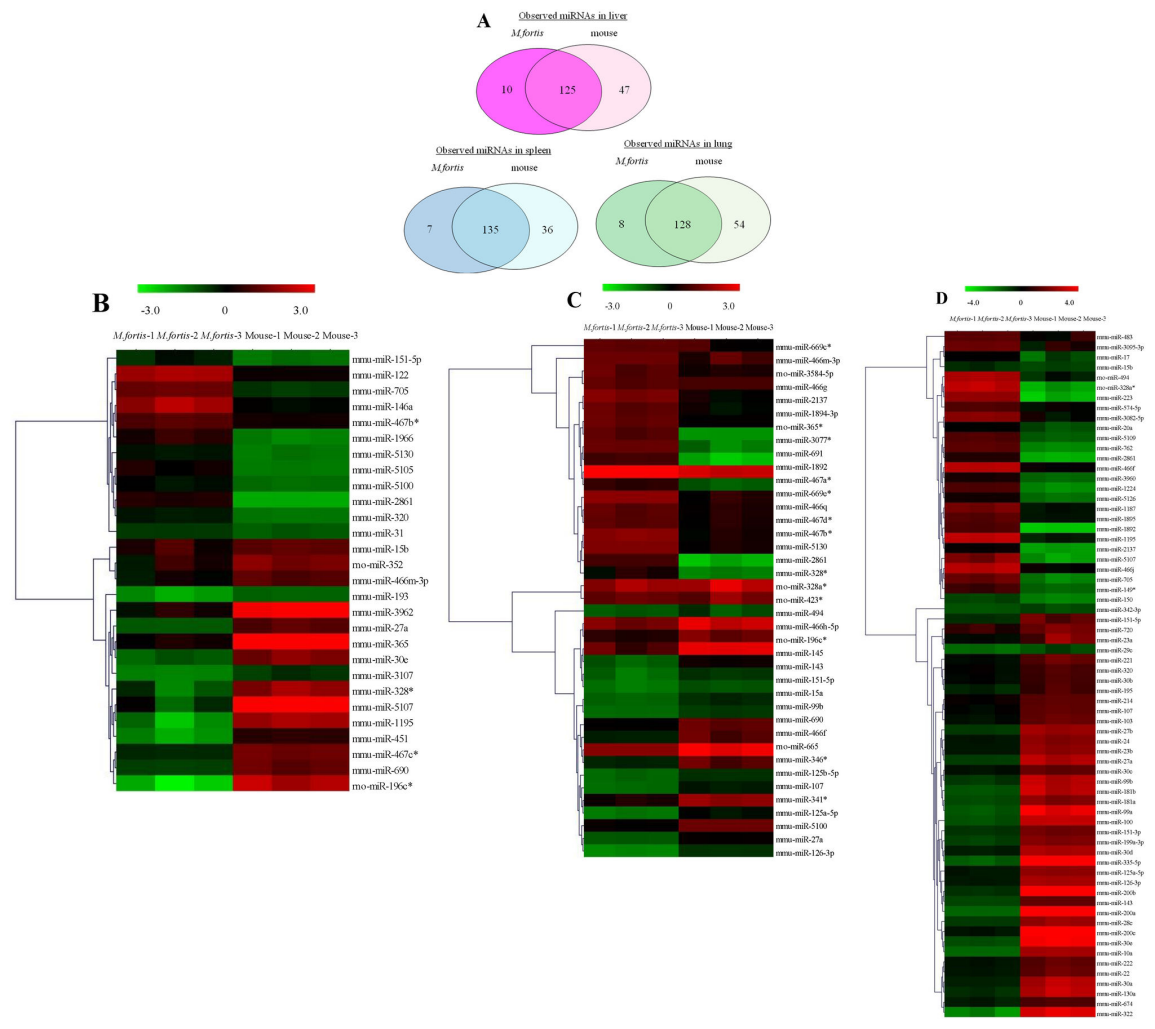
Expression profiling of miRNAs in different tissues of *M. fortis* and mice following *S. japonicum* infection. A:Comparison of observed miRNA in liver, spleen and lung of *M. fortis* and mice; B-D: miRNA expression profile in different tissue of *M.fortis* and mice following *S.japonicum* infection. The left and right panel shows a heat-map of selected miRNAs that showed changes in expression in different tissue in *M. fortis* and mice following *S. japonicum* infection. B: differentially expressed miRNA in liver, C: differentially expressed miRNA in spleen, D: differentially expressed miRNA in lung. Clustering of the microarray showing the stastically significant(*p<0.05) miRNAs in different tissues of *M. fortis* compared with mice. Three replicates from each tissue cluster together. Both up-regulated (the red color ) and down-regulated (the green color) miRNAs from the mean were identified. Then Columns and rows represent samples and particular miRNAs.

The expression profiles of 1777 mature miRNAs were assessed using miRNA microarrays (Sanger miRbase v17.0) in the liver, spleen and lung of *M. fortis* and BALB/c mice in uninfected and *S. japonicum*-infected groups. Excluding any miRNAs with a signal intensity of <500, differences in miRNAs expressed in the infected compared with the uninfected animals are shown in Figure 2A. In total, 135 (172) miRNAs in liver, 142 (171) miRNAs in spleen and 136 (182) miRNAs in lung of *M. fortis* (mice) were identified. Among these, 125, 135 and 128 miRNAs (Totally 162 miRNAs) were common to both species in liver, spleen and lung, respectively. The remainder might be unique differentially expressed miRNAs in the tissues tested from each set of animals. A dendrogram of a hierarchical clustering analysis of differentially expressed miRNAs between the two species is shown in Figure 2B,C and D. The result suggests a unique miRNA expression pattern in *M. fortis* under schistosome infection status. Differentially expressed miRNAs in *M. fortis* when compared with mice are mostly listed separately in Table 1 and Table S2, with values of log_2_(infected/control) and -fold changes. A positive log_2_ value indicates upregulation and a negative log_2_ value indicates down-regulation. Among the detectable miRNAs differentially expressed in the *M. fortis* samples, a total of 68 different miRNAs species were characterized, of which 12 were in liver, 32 in spleen and 34 in lung. In addition, miRNAs specifically expressed in *M. fortis* were also detected: 6 in liver (4 of which were up regulated and 2 were down regulated), 5 in spleen and 5 in lung (all of which were down regulated). 

**Table 1:** Examples of differentially expressed miRNAs in different tissues in *M.fortis* compared with mice infected with *S japonicum*.

Tissue	miRNA	*M.fortis*		Mice	Tissue	miRNA	*M.fortis*		Mice	Tissue	miRNA	*M.fortis*		Mice
		Fold change					Fold change					Fold change		
		(infected/uninfected)					(infected/uninfected)					(infected/uninfected)		
	rno-miR-223	18.38				mmu-miR-691	20.68	5.21			rno-miR-223	9.25	0.05	
	mmu-miR-223	8.34				mmu-miR-328*	3.51	4.5			mmu-miR-1195	8.22	0.74	
	rno-miR-365*	4.76				mmu-miR-467a*	3.14	1.33			rno-miR-328a*	8	0.16	
	mmu-miR-122	3.92	1.12			rno-miR-665	3.07	7.36			mmu-miR-466f	7.52	1.07	
	mmu-miR-146a	3.76	0.97			mmu-miR-467d*	3.07	1.17			mmu-miR-466j	7.52	1.01	
	mmu-miR-705	2.39	0.64			mmu-miR-467b*	2.85	1.19			rno-miR-494	6.54	0.68	
	mmu-miR-872	2.19				rno-miR-223	2.73	0.8			mmu-miR-223	4.96	0.14	
	mmu-miR-467b*	2	1.16			mmu-miR-494	2.68	5.43			mmu-miR-705	3.16	0.24	
	mmu-miR-328*	0.5	3.27			mmu-miR-574-3p	2.43	1.69			mmu-miR-483	2.71	1	
Liver	mmu-miR-27a	0.49	2.03			mmu-miR-2137	2.28	1.04			mmu-miR-762	2.69	0.23	
	mmu-miR-30e	0.47	2.77			mmu-miR-466g	2.17	0.97			mmu-miR-1895	2.6	0.81	
	mmu-miR-322	0.38				mmu-miR-669e*	2.14	1.25			mmu-miR-574-5p	2.41	0.97	
	mmu-miR-3107	0.35	0.65		Spleen	mmu-miR-669c*	2.11	1.25		lung	mmu-miR-1224	2.33	0.22	
	mmu-miR-1195	0.32	3.78			mmu-miR-466q	2.1	1.22			mmu-miR-497	0.49		
	mmu-miR-451	0.3	1.42			mmu-miR-466m-3p	2.08	1.79			mmu-miR-150	0.48	0.24	
	mmu-miR-193	0.28	0.45			mmu-miR-27a	0.49	1.06			mmu-miR-143	0.47	2.93	
	rno-miR-196c*	0.19	4.44			mmu-miR-145	0.47	1.15			mmu-miR-181a	0.45	4.23	
	mmu-miR-181c	0.17				mmu-miR-15a	0.44	0.72			mmu-miR-30e	0.43	15.24	
						mmu-miR-107	0.44	0.81			mmu-miR-100	0.42	8.46	
						mmu-miR-125b-5p	0.44	0.68			rno-miR-143	0.41	3.01	
						mmu-miR-99b	0.42	0.63			mmu-miR-181b	0.41	7.78	
						mmu-miR-143	0.41	0.66			mmu-miR-99a	0.4	13.64	
						mmu-miR-125a-5p	0.38	0.9			mmu-miR-375	0.35		
						mmu-miR-151-5p	0.38	0.51			mmu-miR-200a	0.33	71.01	
						mmu-miR-126-3p	0.33	0.67			mmu-miR-10a	0.33	6.02	
						mmu-miR-99a	0.33				mmu-miR-29c	0.31	0.44	
						mmu-miR-10a	0.32				mmu-miR-34c	0.31		
						mmu-miR-100	0.29				mmu-miR-34b-3p	0.25		
						mmu-miR-10b	0.29				mmu-miR-429	0.23		
						mmu-miR-335-5p	0.25				mmu-miR-322	0.23	11.24	

A full list of differentially expressed miRNAs in different tissues in *M.fortis* compared with mice infected with *S japonicum* is shown in Table S2

### Analysis of the biological function of the differentially expressed miRNAs in *M. fortis* under infection status

The main functions of the differentially expressed miRNAs (with the reported function) in the different tissues of *M. fortis* infected with *S. japonicum* are shown in Table 2 (Table S3). Some differentially expressed miRNAs in liver had important functions, such as involvement in nutrient metabolism, including a cholesterol metabolism regulator (miR-122), a lipid metabolism regulator (miR-705), an adipocyte differentiation and regulation factor (miR-27a and miR-193), and erythrocyte differentiation (miR-223 and miR-451). Additionally, there was an up-regulated miRNA miR-146a with a function in the innate immune system associated with monocyte regulation. As shown in Table 2, up-regulated miRNAs in the spleen were mainly related with pro- and anti-apoptotic proteins, but those miRNAs that were down regulated were all involved in immune system regulation, including miR-15a (regulation of lymphoid development), miR-107 (regulation of macrophage adhesion) and miR-125a-5p (regulation of the inflammatory response and lipid uptake). Among the differentially expressed miRNAs in lungs, most were correlated with immune system regulation, including miR-223 (regulation of the immune response), miR-1224 (regulation of tumor necrosis factor), miR-150 (differentiating stem cells towards megakaryocytes and control of B and T cell differentiation), miR-200a (regulation of immune response). Some of the miRNAs were involved in nutrient metabolism, such as miR-705 (regulation of lipid metabolism and inflammation), miR-143 (regulation of adipocyte differentiation) and miR-375 (regulator of glucagon levels and gluconeogenesis).

**Table 2:** Main functions of the differentially expressed miRNAs in different tissues in *M.fortis* infected with *S. japonicum*.

Tissue	miRNA	Function
	Up-regulated	
	mmu-miR-122	regulation of cholesterol metabolism;a regulator of fatty-acid metabolism
	mmu-miR-146a	regulation of Monocyte Functional;regulation of inflammation and function in the innate immune system
	mmu-miR-705	regulation lipid metabolism and inflammation
Liver	mmu-miR-223	promoting granulocytic differentiation and suppression of erythrocytic differentiation
	Down-regulated	
	mmu-miR-27a	negative regulator of adipocyte differentiation
	mmu-miR-451	erythroiddifferentiation;regulates the drug-transporter protein P-glycoprotein
	mmu-miR-193	regulation of adipogenesis
	Up-regulated	
	mmu-miR-494	targeting proapoptotic and antiapoptotic proteins
	Down-regulated	
Spleen	mmu-miR-15a	regulation of lymphoid development
	mmu-miR-107	regulation of macrophage adhesion; regulator of insulin sensitivity
	mmu-miR-125a-5p	regulation of the inflammatory response and lipid uptake
	Up-regulated	
	mmu-miR-223	regulated in immune response;promoting granulocytic differentiation and suppression of erythrocytic differentiation
	mmu-miR-1224	regulation of tumour necrosis factor
	mmu-miR-705	regulation lipid metabolism and inflammation
	Down-regulated	
Lung	mmu-miR-150	differentiating stem cells towards megakaryocytes and control B and T cell differentiation
	mmu-miR-143	regulation of adipocyte Differentiation
	mmu-miR-30e	corresponding gain in Bcl-2 expression and decreases in pro-apoptosis genes
	mmu-miR-200a	regulated in immune response
	mmu-miR-375	regulate glucagon levels and gluconeogenesis
	mmu-miR-34c	part of the p53 tumor suppressor network
	mmu-miR-34b-3p	part of the p53 tumor suppressor network

The full list of the functions of the differentially expressed miRNAs in different tissues infected with *S. japonicum* in *M.fortis* with the related reference is in Table S3.

### Target prediction of differentially expressed miRNAs

**Figure 3:**
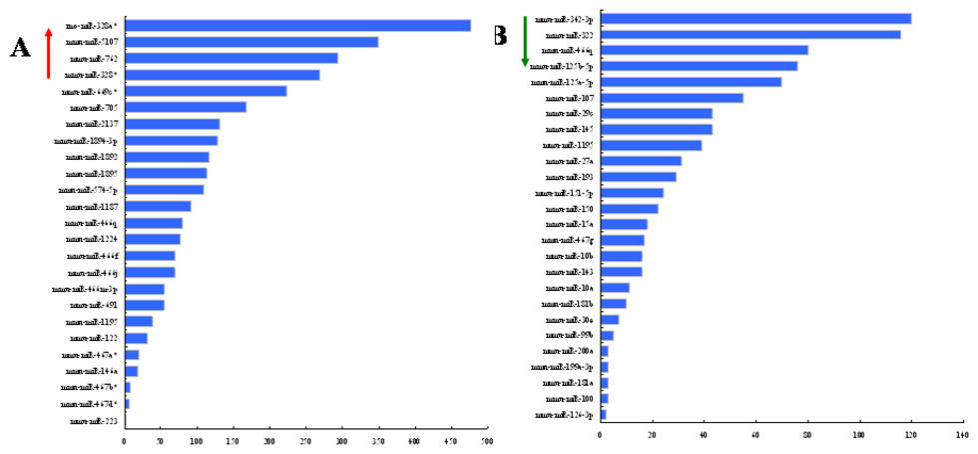
The statistics of predicted target genes for differentially expressed miRNAs in *M. fortis* compared with mice following *S. japonicum* infection. Up-regulated miRNAs in *M. fortis* were listed on the upper chart (A) and down-regulated miRNAs were in the lower chart (B). All the target genes show increased number. The vertical axis is the differentially expressed miRNAs and the horizontal axis is the target gene number.

To identify the target mRNAs of the differentially expressed miRNAs, we performed target prediction for all the differentially expressed miRNAs in the tissues of *M. fortis* and mouse. Of the 68 different miRNA species, target genes were predicted for only 40 of them using the four different online software programs. The quantity of target genes predicted for each differentially expressed miRNA varied from 2 (miR-126-3p) to 477 (miR-328a*), with an average of 138 for up-regulated miRNAs and 34 for down-regulated miRNAs (Figure 3A,B). 

### GO and KEGG pathway analyses of the predicted target genes of differentially expressed miRNAs in *M. fortis*

**Figure 4:**
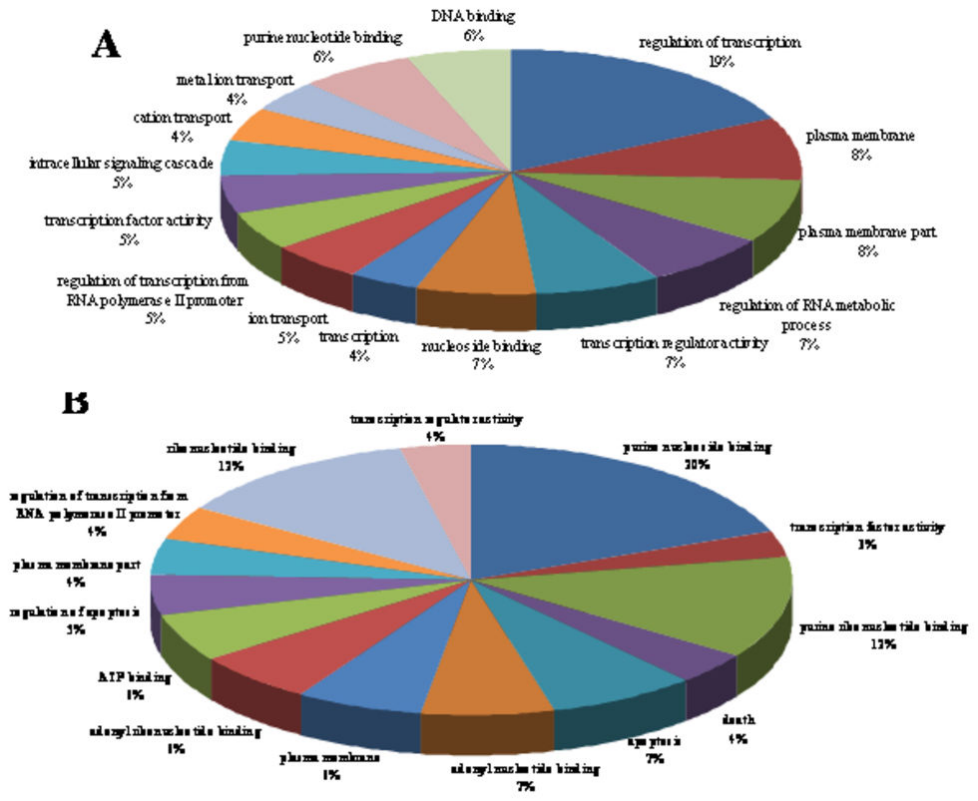
Pie charts showing the distribution of GOs for predicted target genes of differentially expressed miRNAs in *M. fortis* compared with mice following *S. japonicum* infection. A represents the category of the GOs for predicted target genes of miRNAs up-regulated in liver, spleen and lung of *M. fortis*; B represents the category of the GOs for predicted target genes of miRNAs down-regulated in liver, spleen and lung of *M. fortis*. A full list of GO analysis for predicted target genes of differentially expressed miRNAs in *M. fortis* compared with mice infected with *S. japonicum* is shown in Table S4.

To further understand the function of the differentially expressed miRNAs, GO and KEGG pathway analyses were performed on their target genes. David gene annotation was applied to explain the biological effect of miRNAs on the basis of the top 20% of the miRNA targets. The top 15 enrichment GO annotations among the predicted target genes of differentially expressed miRNAs in *M. fortis* were analyzed as shown in Figure 4A,B[29]. GO analyses of these predicted target genes revealed that some of the target genes have important biological functions in the host against the S. *japonicum* infection (Table S4). As shown in Figure 4A, the specific GO of the target genes related to the miRNAs up-regulated in liver, spleen and lung of *M. fortis* that were involved mainly in cellular processes (e.g. DNA binding and regulation of transcription and transcription), signal transduction (e.g. cation transport and intracellular signaling cascades), metabolism (e.g. ion transport, metal ion transport and regulation of RNA metabolic process) , etc.. The specific GO of the target genes related to the down-regulated miRNAs in liver, spleen and lung of *M. fortis* were involved mainly in cellular processes (e.g. adenyl nucleotide binding, ATP binding, purine ribonucleotide binding and ribonucleotide binding), signal transduction (e.g. death, apoptosis and regulation of apoptosis ) , etc.

The KEGG pathway annotation of all the target genes of the miRNAs is shown in Table 3. Among the targets for differentially expressed miRNAs in liver of *M. fortis*, miR-705 and miR-322 had important roles in the immune reaction (involved in the neurotrophin signaling pathway, chemokine signaling pathway and B cell receptor signaling pathway in miR-705) and metabolism (associated with inositol phosphate metabolism and the phosphatidylinositol signaling system in miR-322). Among the targets for differentially expressed miRNAs in spleen of *M. fortis*, miR-328*, miR-10a and miR-10b had important roles in the immune response (involved in Toll-like receptor signaling pathway in miR-10a and miR-10b) and signal pathway induction (involved in MAPK signaling pathway and Wnt signaling pathway in miR-328*). Among the targets for differentially expressed miRNAs in lung of *M. fortis*, miR-466j, miR-322, miR-34c and miR-497 had important roles in immune function (involved in the Toll-like receptor signaling pathway in miR-497), signal pathway induction (involved in the calcium signaling pathway in miR-466j), and nutrition metabolism (involved in inositol phosphate metabolism in miR-322 and the insulin signaling pathway in miR-34c ).

**Table 3:** KEGG pathway of the targets of the differentially expressed miRNAs in different tissue of *M.fortis* following *S.japonicum* infection.

Tissue	miRNA	KEGG pathway	Putative Target	P value
Liver	Up-regulated miRNA			
	mmu-miR-705	Neurotrophin signaling pathway	PDK1, MAPK1, YWHAZ, MAP3K3, GRB2, YWHAB, RHOA, RAF1, MAPKAPK2, TRAF6, FRS2, SHC2	2.39E-06
		MAPK signaling pathway	GRB2, TAOK1, FGF11, RAF1, FGF10, ELK1, MAPKAPK2, STK3, FOS, MAPK1, MAP3K3, PAK2, PRKACA, NFATC4, TRAF6, RASA1	3.35E-05
		Chemokine signaling pathway	CCL1, MAPK1, ADCY1, GRB2, RHOA, RAF1, PRKACA, ADRBK1, GRK5, GNG4, SHC2	2.42E-03
		B cell receptor signaling pathway	FOS, MAPK1, GRB2, RAF1, NFATC4, CD79A	1.82E-02
		Natural killer cell mediated cytotoxicity	MAPK1, TNFRSF10B, GRB2, RAF1, NFATC4, IFNGR2, SHC2	2.91E-02
	rno-miR-365*	Endocytosis	RAB11FIP4, GIT1, RAB5B, PDGFRA, LDLRAP1, AGAP3, EHD4	2.00E-02
	Down-regulated miRNA			
	mmu-miR-27a	MAPK signaling pathway	BRAF, MAPK14, GNA12, CACNG2, DUSP6	2.69E-04
	mmu-miR-322	Inositol phosphate metabolism	INPP5K, PIP5K1B, IPPK	1.34E-02
		Phosphatidylinositol signaling system	INPP5K, PIP5K1B, IPPK	2.50E-02
	Up-regulated miRNA			
Spleen	mmu-miR-328*	MAPK signaling pathway	TAOK1, TGFBR1, RELA, CACNB1, MKNK2, TGFB3, MKNK1, CACNG2, CACNG1, AKT1, DUSP3, MAP3K3, DUSP14, RPS6KA2, PPP3CB, MAPK9, PDGFRB, CACNA1E, RASA1, MAP3K11	3.52E-08
		Cytokine-cytokine receptor interaction	IL9R, TNFRSF10B, OSMR, FLT4, TGFBR1, TGFB3, PDGFRB, EDAR, CD40, BMP7	1.79E-02
		Wnt signaling pathway	WNT1, PPARD, PPP2R5B, CCND2, PPP3CB, MAPK9, FZD6	3.48E-02
		Jak-STAT signaling pathway	AKT1, PTPN6, IL9R, CCND2, OSMR, BCL2L1, SPRY4	3.78E-02
	Down-regulated miRNA			
	mmu-miR-125a-5p	MAPK signaling pathway	RPS6KA1, RAC3, MAP3K8, MAPKAPK2, TRAF6, MAP3K11	4.65E-02
	mmu-miR-10a	Toll-like receptor signaling pathway	IFNAR2, IRF5, MAP2K6, TRAF3	5.48E-04
	mmu-miR-10b	Toll-like receptor signaling pathway	IRF5, MAP2K6, TRAF3	7.71E-03
	Up-regulated miRNA			
Lung	mmu-miR-466j	Calcium signaling pathway	EGFR, ADCY1, CACNA1E, CAMK2A	4.40E-02
	mmu-miR-705	Neurotrophin signaling pathway	PDK1, MAPK1, YWHAZ, MAP3K3, GRB2, YWHAB, RHOA, RAF1, MAPKAPK2, TRAF6, FRS2, SHC2	2.39E-06
		MAPK signaling pathway	GRB2, TAOK1, FGF11, RAF1, FGF10, ELK1, MAPKAPK2, STK3, FOS, MAPK1, MAP3K3, PAK2, PRKACA, NFATC4, TRAF6, RASA1	3.35E-05
		Chemokine signaling pathway	CCL1, MAPK1, ADCY1, GRB2, RHOA, RAF1, PRKACA, ADRBK1, GRK5, GNG4, SHC2	2.42E-03
		B cell receptor signaling pathway	FOS, MAPK1, GRB2, RAF1, NFATC4, CD79A	1.82E-02
		Natural killer cell mediated cytotoxicity	MAPK1, TNFRSF10B, GRB2, RAF1, NFATC4, IFNGR2, SHC2	2.91E-02
	Down-regulated miRNA			
	mmu-miR-322	Inositol phosphate metabolism	INPP5K, PIP5K1B, IPPK	1.34E-02
		Phosphatidylinositol signaling system	INPP5K, PIP5K1B, IPPK	2.50E-02
	mmu-miR-34c	Insulin signaling pathway	RHOQ, MAPK9, MAPK8, PRKACB, LIPE, HRAS1	4.65E-03
		Neurotrophin signaling pathway	PDK1, MAP3K3, MAPK9, MAPK8, HRAS1	1.97E-02
		Cytokine-cytokine receptor interaction	VEGFB, CSF1, TGFB3, PDGFRA, PDGFRB, IL13	4.43E-02
	mmu-miR-497	Toll-like receptor signaling pathway	CD40, TAB1, TRAF6, IFNAR1, TRAF3	2.93E-02
		MAPK signaling pathway	MAP3K3, TAOK1, PPP3CB, TGFB3, CACNA1E, TAB1, TRAF6, RASA1	3.63E-02

### MiRNA–gene network

**Figure 5:**
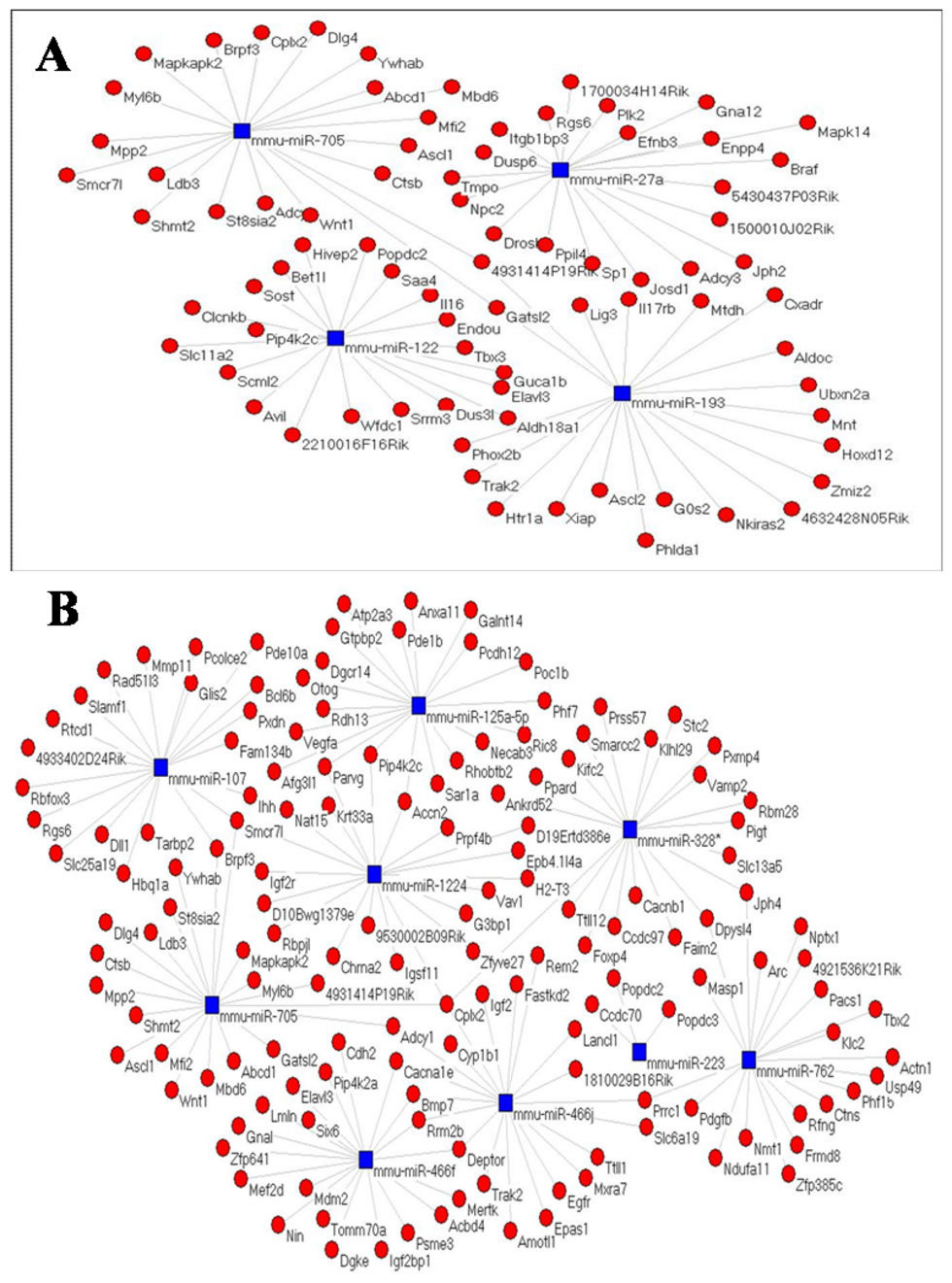
miRNA-target-network. Red cycle nodes represent mRNA and blue box nodes represent miRNA. (A) Significant miRNAs differentially expressed in liver. The left subgraph shows up-regulated miRNA–mRNA network and the right is down-regulated.(B) Significant miRNAs differentially expressed in spleen or lung.The upper subgraph shows under-expression and the lower subgraph is the over-expression microRNA–mRNA network. Mmu-miR-125a-5p and mmu-miR-107 are under-expression miRNAs in spleen. Two miRNAs(mmu-miR-466f, mmu-miR-466j) are the highest degree in lung. Edges represent the inhibitive effect of miRNA on mRNA.

To gain insight into the interactions between miRNAs and their target genes, we carried out an miRNA–gene network analysis to study the relations among the differentially expressed miRNAs in the different tissues from *M. fortis* (Figure 5). Several differentially expressed miRNAs were found with a metabolic function in liver of *M. fortis* (Figure 5A) and with an immune and inflammatory response in spleen and lung of *M. fortis* (Figure 5B). Finally, we established miRNAs regulation networks of schistosome infection of *M. fortis*, which indicated that the differentially expressed miRNA target genes might have important roles in the reactions of the host associated with *S. japonicum* infection. 

### Validation of miRNA microarray data with qPCR analysis

**Figure 6:**
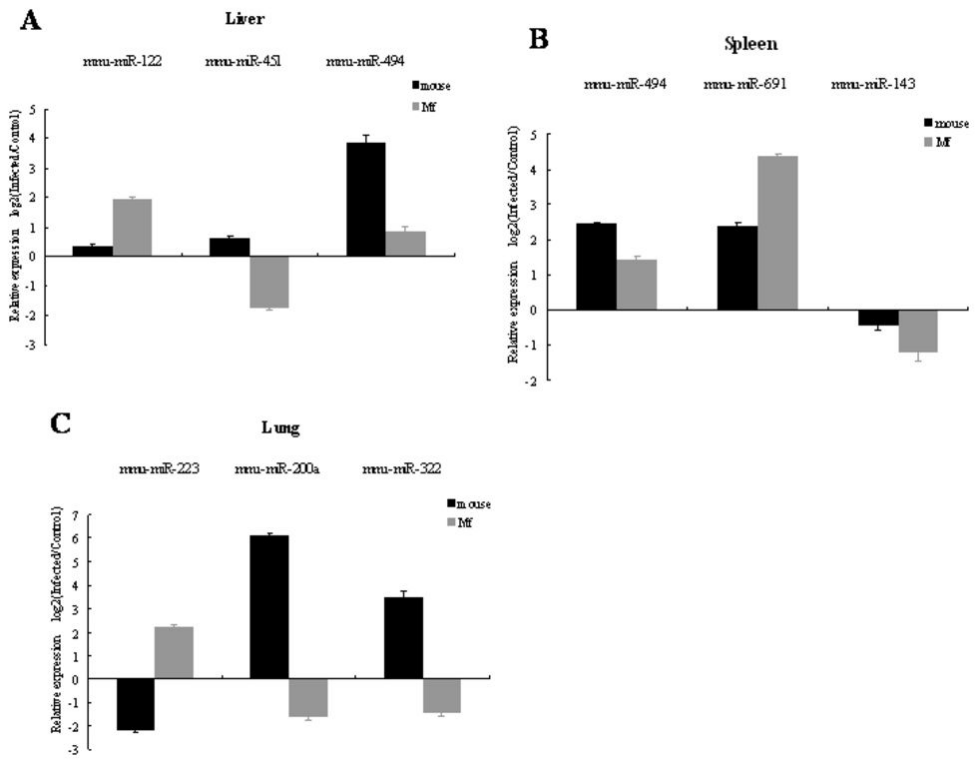
Result of *qPCR* confirmation of miRNA microarray data subset. Selected miRNA expression profile was validated in liver, spleen and lung between *M. fortis* and mice with *qPCR*, respectively. Expression rates between various samples are showed by fold change. The data presents the mean and standard error of the mean derived from triplicate experiments.

Nine selected miRNAs and the housekeeping gene U6 small RNA were assayed by *qPCR* to confirm the results of the expression profiles showed by the microarray platform. MiR-122, miR-451 and miR-494 were detected in liver, miR-494, miR-691 and miR-143 in spleen, and miR-223, miR-200a and miR-322 in lung. The expression patterns validated by *qPCR* correlated well with those of the microarray data (Figure 6, Table S2).

## Discussion

Previous studies had shown that the BALB/c mouse is a susceptible host, whereas *M. fortis* is a non-permissive host for *S. japonicum*. In the latter, most schistosomula were dead by 15 days p.i. and no worms were able to complete their life cycle; in addition, no pathological changes were observed in the lung or liver of schistosome-infected *M. fortis* even after 42 days p.i. Thus, *M. fortis* has been used as a non-permissive animal model for further investigations of the differences at the tissue, cell and gene expression levels between infected and uninfected animals [10]. Differentially expressed genes and proteins have been studied among worms collected from *M. fortis*, rats and mice, in which apoptosis and nutrient metabolism barriers were identified as the main reasons for the growth and development retardation of the schistosomes [32]. The mammalian hosts provide different living environments for the schistosomes and, therefore, more studies are required to increase understanding of the mechanism of the host response against schistosome infection in each environment throughout which the parasites migrate (e.g. lungs, liver and other organs). To date, no studies have analyzed the expression profile differences, regulatory mechanisms and biological functions of the miRNAs of the host following schistosome infection. In the present study, the pathological changes and miRNA expression profiles in different tissues from the non-permissive schistosome host *M. fortis* and susceptible host mice were investigated, and the biological informatics of the specific expression miRNAs were analyzed. In addition, the target genes of differentially expressed miRNAs in *M. fortis* and their function were predicted, which will provide a better understanding of the regulatory function of miRNAs in the mammalian host associated with *S. japonicum* development and the mechanisms of the anti-schistosome response in *M. fortis*. 

The difference in pathological changes of liver, spleen and lung of *M. fortis* before and 10 days p.i. are shown in Figure 1. The host innate immune response to infectious diseases is mediated by epithelial cells and immune cells, such as macrophages, neutrophils and dendritic cells, which is the first line of defence against infection [9]. As shown in our study, a large increase in the number of eosinophils, macrophages, lymphocytes and some neutrophils was observed in big inflammatory infiltrates in the infected livers of *M. fortis*, and a large number of eosinophils and lymphocytes, but only a few neutrophils were observed in small inflammatory infiltrates of *M. fortis*. In addition, the bile canaliculi epithelium cells were proliferated and many eosinophils and lymphocytes existed in small inflammatory infiltrates adjacent to the portal area of *M. fortis*. Some immune reactions, such as an increase in the number of multinucleated giant cells and inflammation in the pleura were observed in mice 10 days p.i. [9], but similar phenomena were not observed in non-infected *M. fortis*. Although progress has been made in understanding the causative mechanisms of the immune response in *M. fortis*, knowledge of the interplay between the regulatory networks of the host and schistosome is still lacking.

To investigate possible mechanisms involved in the anti-schistosome mechanism in *M. fortis*, a genomic-wide analysis of miRNA microarrays applying the miRbase v17.0 was carried out to compare the miRNA expression profile in different tissues in *M. fortis* and mice before and after infection. Many specific miRNAs that were differentially regulated during schistosome infection in the two different hosts were identified:

(i) The function of differentially expressed miRNAs in liver of *M. fortis*

Differentially expressed miRNAs in liver of *M. fortis* are involved mainly in nutrient metabolism, the inflammatory response and cell differentiation. Among them, miR-122, miR-705, miR-193 and miR-27a were related to nutrient metabolism. miR-122, reported as an abundant liver-specific miRNA, showed a significantly high expression level in liver of infected *M. fortis* but no change in spleen and lungs. miR-122 was reported to play an important role to regulate cholesterol and fatty-acid metabolism in the adult liver [33]. miR-705 was reported to be associated with regulation of lipid metabolism and inflammation [34]. miR-193 is a key regulator in adipogenesis [35], and miR-27a is known as an important negative regulator of adipocyte differentiation [36]. The recent annotation of the genome of *S. japonicum* revealed that pathways for fatty acid biosynthesis and metabolism were incomplete and that some enzymes involved in fatty acid degradation and synthesis were important in lipid metabolism [1]. The high expression of miR-122 and miR-705 combined with the downregulation of miR-193 and miR-27a might decrease the levels of fatty acid synthesis and lipid metabolism, which might be important in inhibiting the growth and development of *S. japonicum* in the host environment. miR-451 is a key molecule that regulates erythroid differentiation *in vivo*, and the lack of miR-451 might decrease the hematocrit of mice [37]. The higher expression level of miR-223 in liver of *M. fortis* means a lower differentiation level of erythrocyte, given that miR-223 functions to suppress erythrocytic differentiation [38]. Given that the main food resource of schistosomes is the erythrocyte of the host, the higher expression level of miR-223 and the lower expression level of miR-451 might affect the quality and quantity of the erythrocyte from *M. fortis*, which could in turn reduce the food resource of schistosome. It has been demonstrated that miR-146a mediates protective innate immune tolerance in the bacteria-induced epithelial damage in neonates [39]. It is unknown whether there is a relation between the higher expression level of miR-146a and the innate immune tolerance of *M. fortis* against schistosome, and so further studies are required.

(ii) Effect of the differentially expressed miRNAs in spleen of *M. fortis*

The differentially expressed miRNAs in spleen of *M. fortis* were mainly involved in apoptosis and the immune response. Among four differentially expressed miRNAs, miR-494 was the only one with higher expression in *M. fortis*, with the function of targeting pro- and anti-apoptotic proteins [19]. miR-15a is known to be a regulator in lymphoid development [40]. miR-107 can increase macrophage adhesion via cyclin-dependent kinase 6 by downregulation of Toll-like receptor-4 (TLR4) [41,42,43]. miR-125a-5p can regulate the inflammatory response and lipid uptake in oxLDL-stimulated monocyte-derived macrophages [44]. Antibody-dependent cell-mediated cytotoxicity (ADCC) was used to evaluate the killing effect of the immune response in *M. fortis*, and the results showed that more macrophages and eosinophils from *M. fortis* were binding to the schistosomula *in vivo* [45], which means that the miRNA regulation of the adhesion and development of macrophages and eosinophils from *M. fortis* might be important in killing schistosomes in *M. fortis*. Surprisingly, the four differentially expressed miRNAs in spleen have the same expression trend between the two species, which might mean that the miRNA regulation network in the spleen of both mice and *M. fortis* are similar, but that in *M. fortis* can trigger a stronger immune reaction against *S. japonicum* infection.

(iii) Effect of differentially expressed miRNAs in lungs of *M. fortis*

The differentially expressed miRNAs in lung of *M. fortis* were mainly involved in nutrient metabolism, immune regulation and apoptosis. miR-223 was identified as a regulator [46] and can be dynamically regulated during T and B cell development, involved in immune response [47]. miR-1224, with the function as: ‘negative regulator of tumour necrosis factor’, which has been shown to regulate tumour necrosis factor-α (TNF-α) gene expression by modulating Sp1, might be involved in regulating the LPS-mediated inflammatory responses [48]. miR-150 was characterized as having a key role in differentiating stem cells towards megakaryocytes and controlling B and T cell differentiation [40]. miR-200a, miR-34c and miR-34b-3p were identified as regulators of the immune response and part of the p53 tumor suppressor network, respectively [49]. Among the six miRNAs mentioned above (except miR-150), the relative expression levels were significantly different from the related miRNAs in mice, which suggested that the lung of *M. fortis* is one of the main organs in which schistosomula are killed. This result again confirmed that the lung of the host can induce an innate immune response against migrating schistosomula [9]. miR-143 was reported as a regulator of adipocyte differentiation and was significantly down-regulated compared with mice [50,51]. miR-375 was also shown to be a key regulator of glucagon levels and gluconeogenesis and it was also significantly down regulated [52]. As mentioned above, many nutritional components required in the schistosome life cycle are absorbed from the host; therefore, the down regulation of lipid metabolism and carbohydrate metabolism might be one of the key mechanisms to affect the growth and development of *S. japonicum* in *M. fortis*. miR-30 was reported to result in an increase in Bcl-2 expression and a decrease in pro-apoptosis genes, such as Bax, and cleavage of caspases [53,54]. miR-494 and miR-30e were both targets on the apoptosis-related gene involved in the regulation of the level of apoptosis in the host; apoptosis is one of the main reasons for the development and growth retardation of *S. japonicum* in *M. fortis* [55], which means that it might be also one of the key players in the interaction of *S. japonicum* and *M. fortis*.

The major difficulty for understanding the biological functions of miRNAs is determining their specific target genes at the post-transcriptional level. We have predicted the target genes and functions of the differentially expressed miRNAs in *M. fortis* by using bioinformatics analysis. GO analyses revealed that some of the target genes have important biological functions in the host associated with *S. japonicum* development, and that some of these genes in *M. fortis* are involved mainly in cellular processes, signal transduction and metabolism. These results strongly suggest the potential role of miRNAs in the pathogenesis of schistosome infection in *M. fortis* and confirm the importance of bioinformatics in exploring miRNA function. The KEGG pathway analysis of the target genes of the differentially expressed miRNAs showed the genes to be mainly involved in immune-related pathways, such as the Toll-like receptor signaling pathway, cytokine–cytokine receptor interactions and B cell receptor signaling pathways; in signal induction pathways, such as the calcium-signaling pathway, phosphatidylinositol-signaling system, MAPK-signaling pathway and Wnt-signaling pathway; and in nutrient metabolism, such as inositol phosphate metabolism and the insulin signaling pathway. Also, many of the target genes were focused on the immune response-related pathways, such as the Toll-like receptor signaling pathway, cytokine–cytokine receptor interactions and B cell receptor signaling pathways, which means that the immune response toward *S. japonicum* infection might be one of the key players in the defense against infection by this parasite in *M. fortis*. The results illustrate that the mechanism involved in the innate anti-schistosome feature of *M. fortis* are closely related to the repression of nutritional components required by the parasite and a strong host immune response, which are concordant with previous reports[56].

## Conclusions

In summary, the present study revealed substantially different histological changes between tissues of the non-permissive host *M. fortis* and susceptible host mice post *S. japonicum* infection, and a remarkable inverse miRNA profile was revealed for nutritional components metabolism, immune defense diverse hints at a closely crosstalk between miRNAs and host reaction to the schistosome infection. The data in this study could provide new insights into the general mechanisms of infection regulation in *M. fortis*, and could be used to exploit the potential miRNA-regulated networks and the interaction between parasites and different hosts. However, further investigations are required to evaluate the functional consequences of these findings and the biochemical function of the differentially expressed miRNAs.

## Supporting Information

Table S1 **Sequences of the primers used for stem-loop RT-PCR.**
(XLS)

Table S2 **miRNAs expression profile in different tissues of *M.fortis* and mice before and 10 days post infected with *S.japonicum*.**
(XLS)

Table S3 **Main functions of the differentially expressed miRNAs in different tissues infected with *S. japonicum* in *M.fortis*.**
(XLS)

Table S4 **GO analysis for predicted target genes of differentially expressed miRNAs in *M.fortis* compared with Mice following *S.japonicum* infection.**
(XLS)
